# Characterization of pulverized Marula seed husk and its potential for the sequestration of methylene blue from aqueous solution

**DOI:** 10.1186/s13065-019-0530-x

**Published:** 2019-01-30

**Authors:** Joshua N. Edokpayi, Stanley S. Ndlovu, John O. Odiyo

**Affiliations:** 1 Hydrology and Water Resources Department, University of Venda, Thohoyandou, 0950 South Africa; 2 Ecology and Resource Management, University of Venda, Thohoyandou, 0950 South Africa

**Keywords:** Adsorption, Equilibrium, Kinetics, Marula seed husk, Methylene blue, Thermodynamics

## Abstract

Dyes are ranked as an important class of pollutants which affect the aesthetic property of the environment when present even in very low concentrations. This study was carried out to explore the potential use of an agricultural waste (Marula seed husk) to decontaminate methylene blue (MB) from aqueous solution. The effect of change in water chemistry was also examined. The influence of basic adsorption parameters such as contact time, temperature, dosage, pH and particle size on the efficiency of adsorption were investigated. Langmuir and Freundlich isotherms were used to describe the equilibrium data while Pseudo first, second order and Elovich kinetic models were used to evaluate the kinetics of the adsorption process. Thermodynamic parameters such as change in enthalpy (ΔH°), entropy (ΔS°) and Gibbs free energy (ΔG°) were evaluated. Natural surface water showed higher MB removal efficiency than de-ionized water. The sorption process was favored more in alkaline pH range (7–10). The dye adsorption process was found to be endothermic, while Δ*G*° was negative implying that the reaction is spontaneous. Functional group analyses on the adsorbent showed the presence of hydroxyl, carbonyl and carboxyl groups. The Langmuir equilibrium model best described the adsorption process based on the linearized coefficient. The Pseudo second order model best described the kinetics of the reaction.

## Introduction

Water is very vital to the sustenance of life on earth. The quality of water has a direct impact on the water use potential [1]. Several pollutants of fresh water sources have been documented [2–4]. The quest for more colorful fabrics has increased the quantity of dyes produced worldwide. The release of dyes containing effluent into surface water body affects the aesthetic property of water even at very low concentrations [5, 6]. The use of dyes is not restricted to textile industries. The release of dye containing materials into the aquatic ecosystem can lead to poor functioning of aquatic biota. There is reduction of light penetration into the bottom of water bodies due to the presence of dye molecules [5, 7, 8]. Most dyes are persistent in nature, toxic and can affect the reproductive potential of aquatic organisms.

Methylene blue (MB) has been reported for use in medicine, food industries, pharmaceutical industry, pulp and paper industries as well as textile industries. Long term exposure to MB has been implicated in chest pain, confusion, anemia, nausea and vomiting, high fever, headache, blue skin and hypertension [9, 10].

Various methods have been reported for the decontamination of dyes from aqueous solution. Most of these methods can be classified as physical, chemical or biological depending on the nature of decontamination. Commonly reported methods for MB removal include coagulation, flocculation, filtration (microfiltration, ultrafiltration and nanofiltration), reverse osmosis, oxidation using various oxidants (chemicals or Ultraviolet rays) and adsorption [11–13].

Adsorption technique has gained increased attention due to the simplicity and ease of application. The most commonly utilized adsorption method involves the use of commercially produced activated carbon. The trained expertise required and high cost of activated carbon are major drawbacks to its extensive application for the sequestration of dyes [11, 12]. A lot of locally available plant materials have been reported useful for the removal of dyes from aqueous solution based on adsorption technology. These include papaya leaves and seeds [14, 15], groundnut shell powder [16], fallen leaves of Platanus [17], pumpkin seed hull [18], rubber seed shell [19–21], walnut sawdust [22], melon seed shell [23], tomato seed [24] and white pine sawdust [25].

Marula plants are common in semi-arid regions in sub-Saharan Africa [26]. Marula fruits and seed have wide domestic and industrial applications such as for making cooking oils, jam, jelly and are used as food. Some parts of the plant have medicinal value for treatment of diseases.

Due to the extensive use of Marula fruit and seed, the seed husk is often discarded as an agricultural waste material which causes environmental pollution. This study seeks to characterize this waste material and investigate its potential use for the sequestration of MB from aqueous solution. The effect of acid and base modification of the adsorbent is presented as well as the effects of change in water chemistry on the sorption process. The equilibrium, kinetics and thermodynamics of the adsorption process are discussed.

## Experimental

### Chemicals and reagents

Analytical grade reagents were used in this study. Methylene blue was purchased from Fischer Scientific (USA). NaOH and HCl were purchased from Merck (Pty) Ltd, South Africa, and were used to adjust the pH of the solution.

### Preparation of adsorbent

**Fig. 1:**
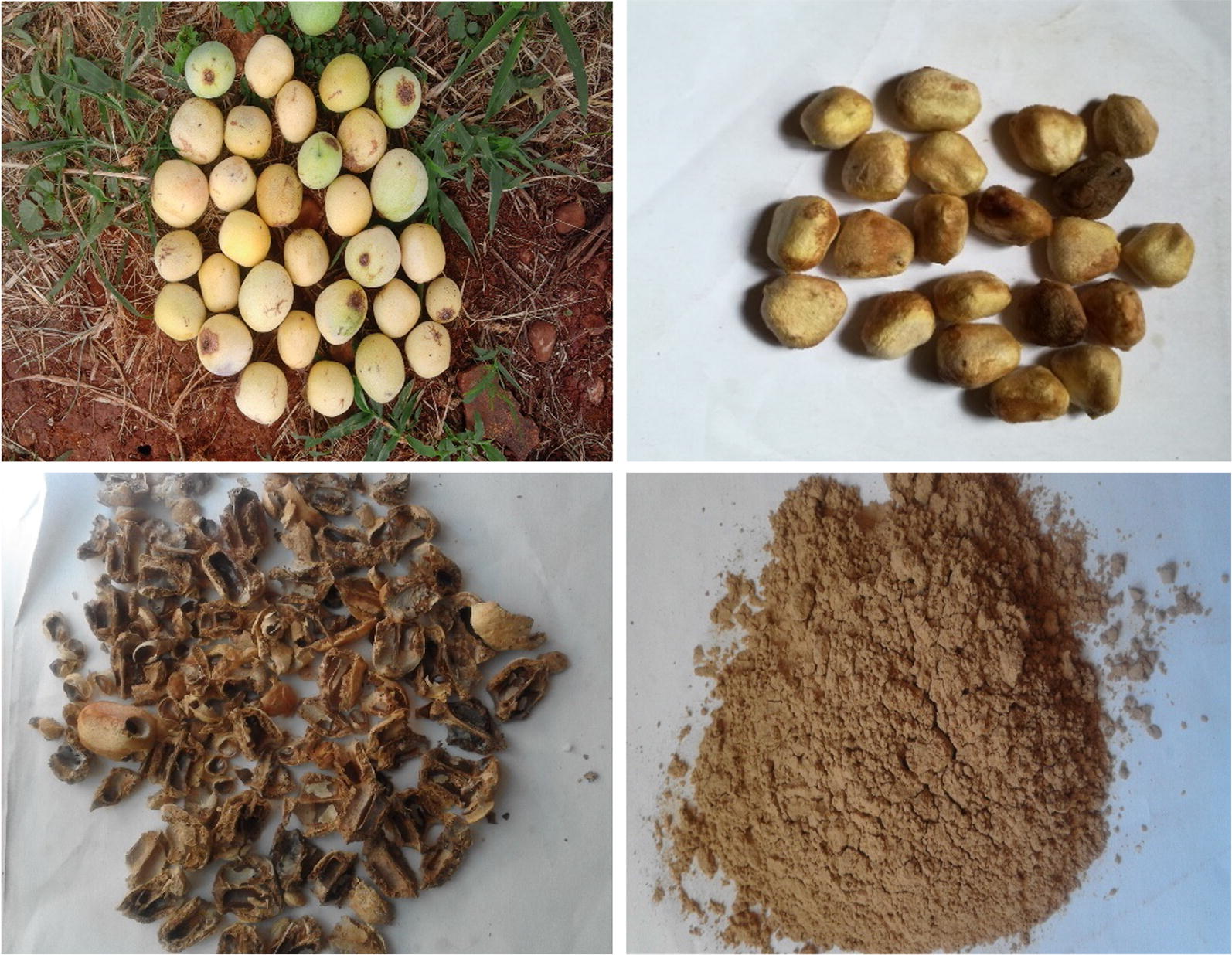
Stages of adsorbent preparation: top left is the Marula fruits, top right is the Marula seeds, down left is the shell of the Marula seed while down right is the pulverized Marula seed husk

Marula fruits were collected at N’wamitwa village in Limpopo province of South Africa. The seeds were removed from the fruits and washed several times with tap water before oven drying (Eco Therm, Labotec) for 12 h at 65 °C. Thereafter, Marula seeds were crushed to obtain seeds husk which were subsequently milled using a Retsch RS 200 pulverizer. The ground material was sieved with a King-Test VB 200/300 sieve shaker to obtain powder of the following range: < 125 and > 250 µm. The process of adsorbent preparation is shown in Fig. 1.

### Preparation of dye solution

A stock solution of 1000 mg/L of MB was prepared by dissolving 0.25 g in 250 mL of de-ionized water. Various working concentrations were prepared from the stock solution. The wavelength of maximum MB absorption ($\lambda_{max}$) was determined by running the solution in a UV–Vis spectrophotometer (Orion Aquamate 7000, Thermoscientific) from 400 to 1000 nm. The $\lambda_{max}$ was determined to be 664 nm. Calibration curve was prepared using 0–50 mg/L of MB solution. A straight line with correlation coefficient of 0.999 was obtained. Dilution was performed for MB concentration > 50 mg/L.

### Characterization of the adsorbent

Fourier transform infrared (FT-IR) spectra of the Marula seed husk were obtained using a Perkin Elmer 100 FT-IR (Waltham, MA, USA) with accessories. The spectra were scanned over the wave number range of 4500 to 400 cm^−1^. An SDT Q600 TGA–DSC analyzer was used to monitor the degradation pattern of the adsorbent. The sample (10 mg) was heated from room temperature (25 °C) to 1100 °C, at a rate of 10 °C/min.

### XRD analysis

0.5 g of the adsorbent were mounted on a sample holder. This was subsequently placed on an X-Ray Diffractometer (BRUKER AXS, Germany). The instrument operates according to a Bragg–Brentano geometry scanning from 5 to 80° in two theta with a step size of 0.0034 deg/2theta. The position sensitive detector records the intensity. The 192 channels of the silicon strip detector give an effective measurement time of 96 s/step. The data was analysed using the International Center from Diffraction Data (ICDD) database to identify the major phases present in the material.

#### Surface morphology of the adsorbent

The samples were loaded into a Zeiss EVO Scanning Electron Microscope (Carl Zeiss Microscopy, Munchen, Germany) at the Electron Microbeam Unit of Stellenbosch University’s Central Analytical Facility (CAF). Zeiss InLens SE (Secondary Electron) and SE2 detectors, as well as Backscatter Electron (BSE) Detector and Zeiss Smart SEM software were used to generate images. For Secondary Electron detection, operating conditions of 3 kV accelerating voltage and 100 pA beam current with a working distance of 3.8–4 mm were used to generate images. For Backscatter Electron detection (BSE), operating conditions of 20 kV accelerating voltage and 11 nA beam current with a working distance of 9.5 mm, were applied. Images were captured in random areas and at a range of magnifications, to characterize grain morphology.

#### Determination of point of zero charge (PZC)

The point of zero charge was investigated using 0.1 M KCl solution. The solutions (40 mL) were transferred into different 100 mL polyethylene bottles. The pH of the solutions were varied between 2 and 11 with 0.1 M HCl and 0.1 M NaOH. 0.1 g of the adsorbent was then added to each of the sample which were equilibrated for 24 h using a thermostated shaking water bath. The samples were centrifuged at 250 rpm for 15 min and the pH of the supernatant was measured. The point of zero charge was estimated by plotting change in pH (∆pH = pHi − pHf) versus initial pH. The point of intercession on the x-axis indicated the point of zero charge of the adsorbent.

#### Modification of the adsorbent

0.5 M of HCl and NaOH were prepared and used for the modification of the adsorbent. The adsorbent was treated with 0.5 M HCl and NaOH solution in a conical flask for 12 h. The samples were filtered and dried in the oven for 12 h at 90 °C. Then, the adsorbent was collected from the oven and kept for further use.

### Effect of time on adsorption efficiency

Two different masses of adsorbent (0.05 g and 0.15 g) were used for this experiment. Each adsorbent mass was poured into a separate flask containing 40 mL of 30 mg/L MB solution. The mixture was taken to a temperature-controlled water bath equipped with a mechanical shaker (EcoBath, Labotec) set at 30 °C for 5–240 min. After 5 min, the first set of mixture was taken out and poured into a centrifuge tube. The samples were centrifuged (LMC, 300, Grant-bio) at 2400 rpm for 10 min. The supernatant was placed in a cuvette and ran in the UV–Vis spectrometer. The same process was repeated after time intervals of 20, 30, 90, 180, 210 and 240 min. The percentage of MB removed at each time point were calculated using the relation in Eq. (1) and the quantity of MB adsorbed was calculated using Eq. (2)

1 $$Percentage\;MB\;removal = \frac{{C_{i} - C_{f} }}{{C_{i} }}100$$

2 $$q_{e} = \frac{{C_{i} - C_{f} }}{W}v$$

where C_i_ and C_f_ (mg/L) are the initial MB concentration and the concentration at equilibrium, q_e_ is the quantity of MB adsorbed, V is the volume of the solution (L) and W is the mass of adsorbent (g).

Similar procedure was followed in assessing the effects of other experimental parameters. Briefly: Different adsorbent dosage in the range of 0.01–0.15 g  were used to study the effects of change in adsorbent dosages, while, particle sizes of < 125 µm and > 250 µm were employed to assess their effects on the adsorption experiments. The influence of pH on the adsorption process was monitored in the pH range of 2–10. The effects of temperature were performed in the range of 313–363 K.

Matrix effects were investigated using natural surface water collected from Mutale River (in Limpopo Province, South Africa) to examine the effects of change in water chemistry on the removal of MB by Marula seeds husk. The natural surface water and de-ionized water were used to prepare 30, 50 and 70 mg/L of MB solutions. The removal efficiency of the adsorbent was compared for both types of water.

## Results and discussion

### Characterization of the adsorbent

**Fig. 2:**
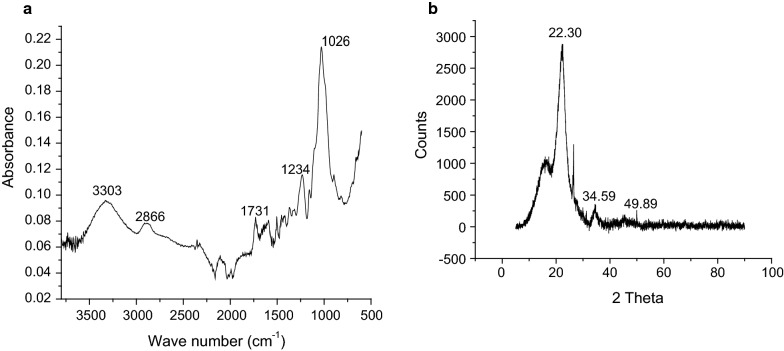
FT-IR and XRD plot of pulverized Marula seed husk

The results from the Fourier transform infra-red showed a broad peak at 3303 cm^−1^ with a high transmittance frequency (Fig. 2a), which can be attributed to the presence of hydroxyl group [27, 28]. The band detected at 2866 cm^−1^ is due to C–H stretching vibrations of alkanes. A medium, weak band recorded at 1731 cm^−1^ corresponds to C=O stretch of carbonyl group [27]. The bands observed at 1234 and 1026 cm^−1^ can be attributed to C–O–C stretching vibrations of ether. The XRD spectra (Fig. 2b) shows a major peak at 22.3° (2θ) with other minor peaks which can be attributed to the presence of cellulosic content of the adsorbent. This is expected as the material is basically of plant origin.

**Fig. 3:**
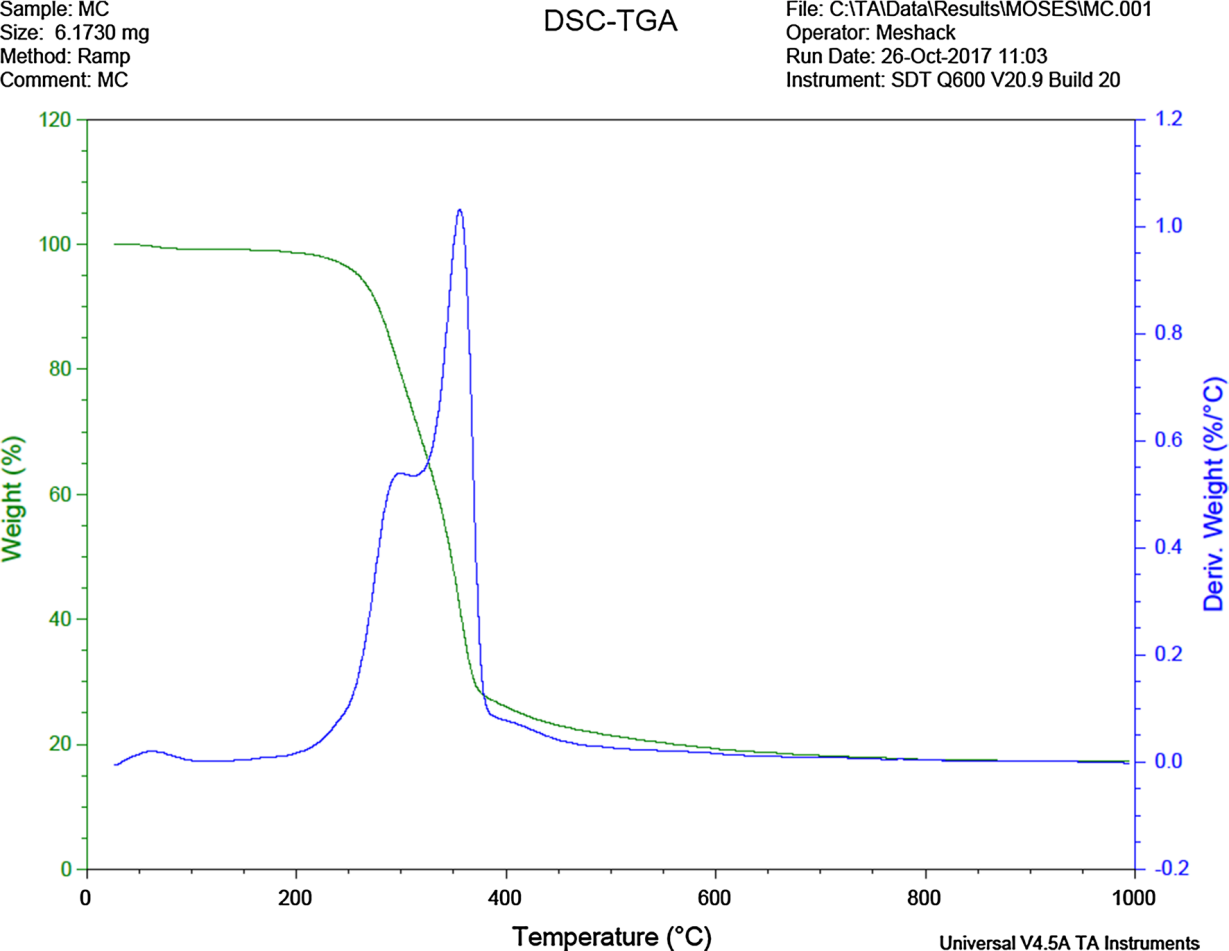
DSC-TGA degradation pattern of pulverized Marula seed husk

Figure 3 shows the results from the thermogravimetry analysis (TGA) and differential scanning colorimetry (DSC) trend of the adsorbent. The initial loss in weight of the adsorbent was recorded at approximately 195 °C, this can be attributed to evaporation of bound water and moisture in the adsorbent [27]. A subsequent loss in weight was detected at 250 °C, due to the thermal degradation of cellulose and hemicellulose in the plant-based material. The final weight loss occured at approximately 370 °C, and could be ascribed to the degradation of lignin, which has a much higher thermal stability than either cellulose or hemicellulose polymers [29]. The corresponding DSC degradation pattern of the adsorbent is also presented in Fig. 3.

**Fig. 4:**
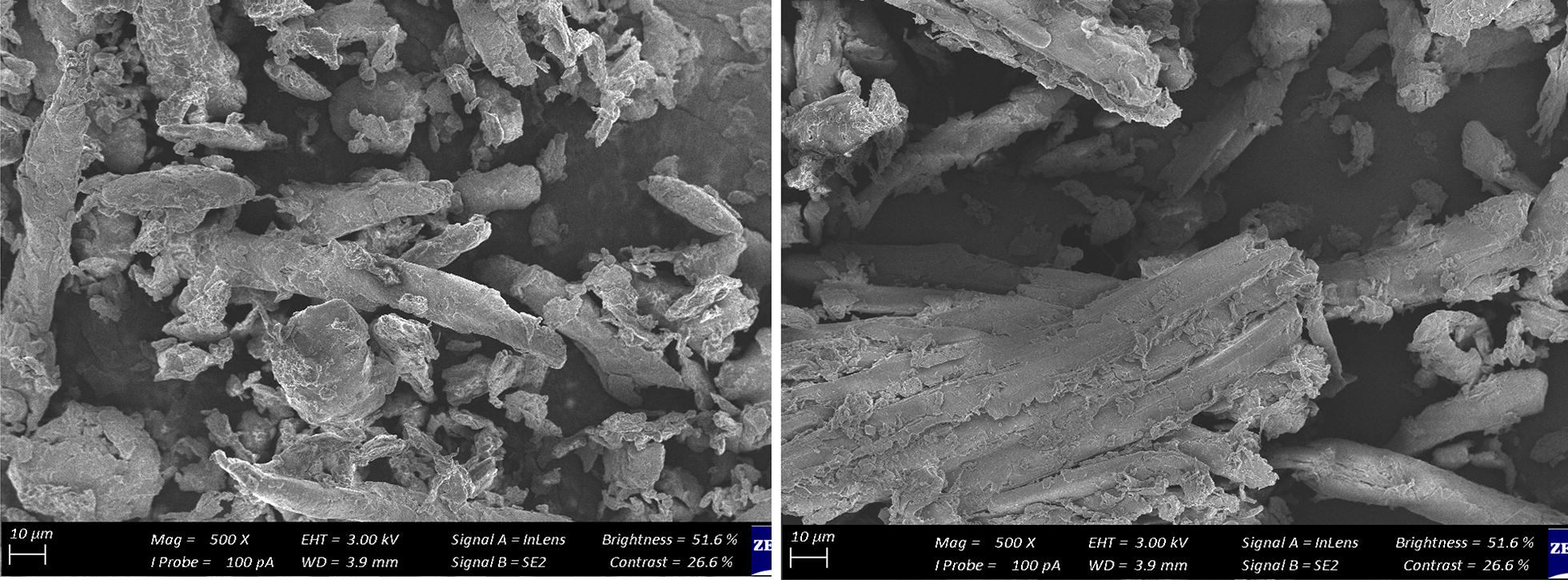
SEM micrograph of raw and spent adsorbent

The surface morphologies of the unused (Fig. 4a) and spent (Fig. 4b) adsorbent is presented in Fig. 4. The raw adsorbent has a lot of cracks and voids with coarse surface suitable for the adsorption of contaminants. The spent adsorbent showed a reduction in the heterogeneous nature of the adsorbent which is a reflection that adsorption had occurred, with the adsorbate attached to the adsorbent.

### Effects of adsorbent modification by acid and base

**Fig. 5:**
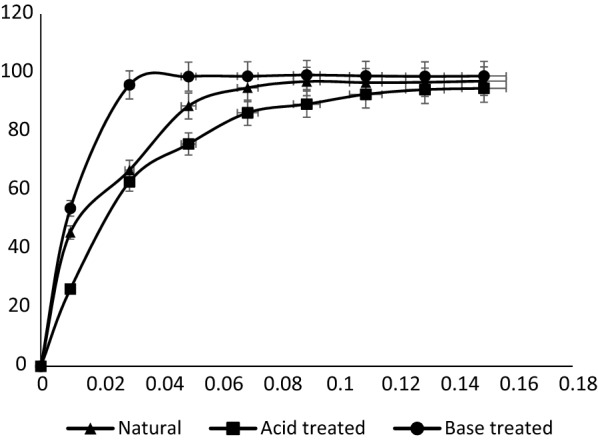
Effects of acid and base modification of the adsorbent

Figure 5 shows the comparative removal efficiency of the natural, acid-treated and base-treated pulverized Marula seed husk for the sequestration of MB. The base-treated Marula seed husk recorded a higher MB removal efficiency than the untreated and acid treated forms. Low adsorbent dosage of the base-adsorbent recorded a higher removal efficiency of MB compared to others. However, at a slightly higher dosage no significant difference was obtained for the various forms of the adsorbents.

### Effect of contact time

**Fig. 6:**
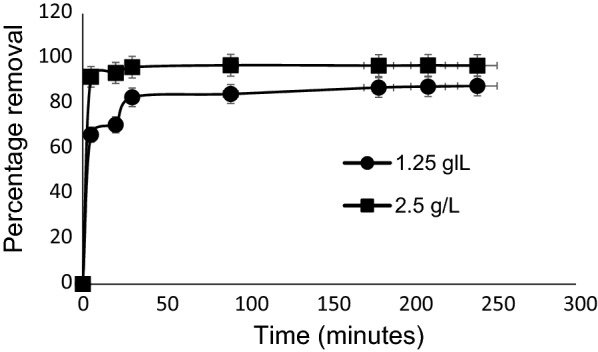
Effects of time on the uptake of MB by pulverized Marula seed husk

Time of equilibration usually plays a major role in adsorption experiments. In this study, there was an initial rapid uptake of MB by Marula seed husk within 5 min of equilibration; 1.25 g/L recorded 66% removal while 2.5 g/L recorded 94% removal (Fig. 6). There was however, a slight increase in MB removal efficiency with increased time from 71% at 20 min to 83% at 30 min with 1.25 g/L. Similarly, a slight increase was also recorded with 2.5 g/L from 94% (20 min) to 96% (30 min). After 30 min, only a slight increase was observed for both dosages.

The initial rapid uptake of MB by pulverized Marula seed husk can be attributed to the presence of more surface area on the adsorbent available for dye adsorption. After the filling of the surfaces, only a few uptakes of the dye removal were observed due to few active sites on the surface of sorbent. This continued until equilibrium was reached where no further increase was recorded. The findings obtained in this study are in consonance with results obtained by other scholars [30–32].

### Effect of adsorbent dosage and concentration

**Fig. 7:**
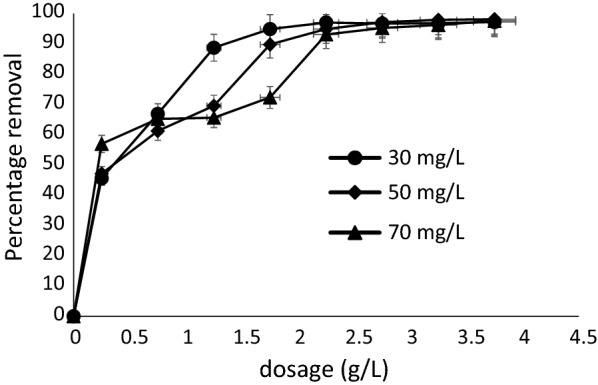
Effects of dosage plot

Figure 7 shows the effects of adsorbent dosage and initial MB concentration on the uptake of MB (30, 50 and 70 mg/L) by 0.25 g/L–4.0 g/L of Marula seed husk. The percentage removal of MB increases with increased adsorbent dosage. From 45% (0.25 g/L) to 98% (4.0 g/L) for 30 mg/L, 47% (0.25 g/L) to 97% (4.0 g/L) for 50 mg/L and 57% (0.25 g/L) to 96% (4.0 g/L) for 70 mg/L, respectively. Generally, higher removal of MB as expected was recorded for 30 mg/L compared to 50 mg/L and 70 mg/L but from 2.5 g/L similar levels of MB were removed by the adsorbent irrespective of the dye initial concentration.

The initial increase in performance with increase in adsorbent dosage is due to the corresponding increase in surface area available for adsorption. The subsequent little additional MB removal recorded could result from either aggregation or overlapping of adsorption sites. No removal was recorded after attainment of equilibrium.

### Effect of particle size

**Fig. 8:**
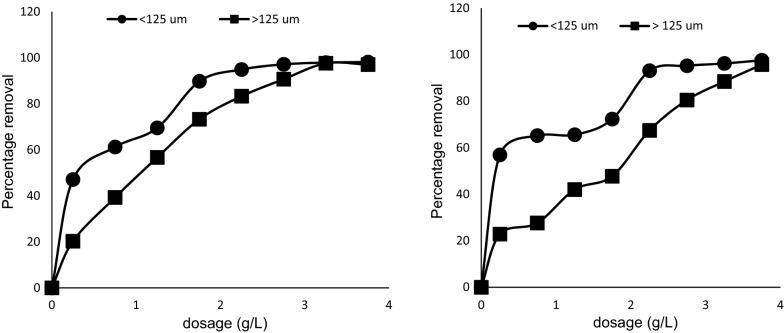
Effects of particle size on the adsorption of MB onto pulverized Marula seed husk. The right is for 50 mg/L of MB while the left is for 70 mg/L of MB

Particle size is known to influence the adsorption rate of many adsorption systems. The small adsorbent particles usually have higher surface area than the larger ones, and this often contributes to more available adsorption active sites, hence resulting in higher adsorption [33]. In this study (Fig. 8), the smaller particle size (< 125 µm) achieved a better adsorption of MB than the larger size (> 125 µm). This could also be due to a reduction in the limitation of internal diffusion and mass transfer of the adsorbate into the adsorbent with smaller particle sizes [34].

### Effect of pH

**Fig. 9:**
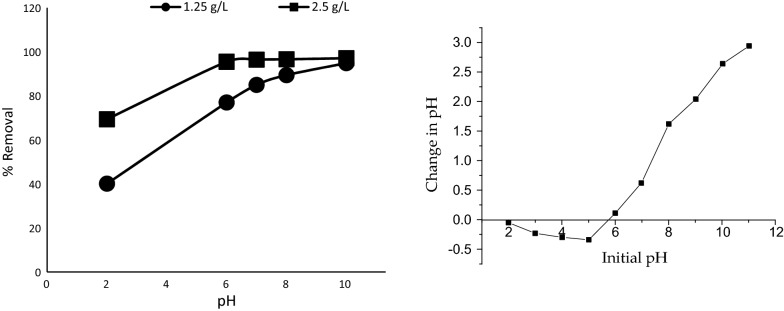
Effects of pH on MB uptake by pulverized Marula seed husk and determination of pH_PHZ_

The effect of pH on MB removal was examined over a range of pH values from 2 to 10 and the results are presented in Fig. 9a. MB removal was minimum at pH of 2 for both dosages of Marula seed husk. There was a significant increase of MB uptake up to pH 6, after which slight increases were recorded up to pH 10. This could be due to increased electrostatic interaction between the dye molecules and the adsorbent at higher pH values [35]. The results imply that percentage removal of methylene blue by Marula seed husk was lower in acidic medium. This might be due to the presence of positively charged hydrogen ions which compete and/or interfere with dye cations for the available adsorption sites [7, 36]. A similar pH trend has been reported by Oden and Ozdemir [37] but disagrees with the study conducted by Jirekar et al. [38] which showed a maximum removal of MB in the acidic pH range.

The point of zero charge of 5.8 was determined for the adsorbent (Fig. 9b). At pH < pH_PZC_, the adsorbent surface is positively charged with high concentrations of H^+^ capable of competing with MB cations for the unadsorbed sites leading to a decrease in the uptake of MB. But when pH > pH_PZC_, the adsorbent surface becomes negatively charged and favors the adsorption of MB due to increased electrostatic force of attraction and decreased H^+^. This result clearly supports the data obtained for the effects of change in pH where low amount of MB was adsorbed at low pH but as the pH increased, significant increase in the uptake of MB was recorded. The pH_PHZ_ obtained in this study (5.8) is slightly lower than that reported for Aleutites Moluccane seeds (5.84) [29] but was higher than the pH_PHZ_ determined for modified celery (4.7) [39] and acid washed black cumin seed (2.0) [40].

### Effect of matrix

**Fig. 10:**
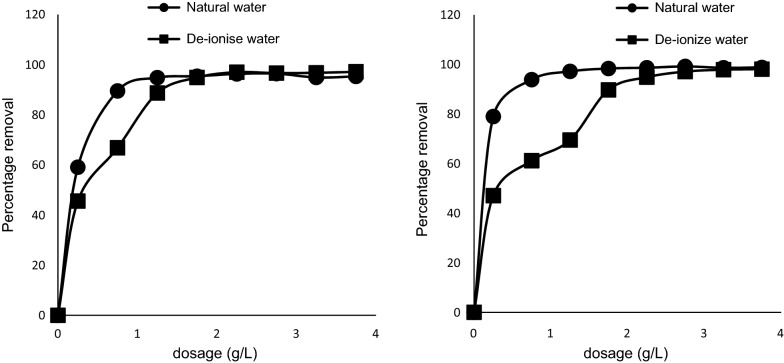
Effects of Matrix on MB uptake by pulverized Marula seed husk. Left is 50 mg/L of MB while right is 70 mg/L

This experiment was performed to determine if change in water chemistry had any influence on the adsorption process. It was established that the adsorption process performed better in natural surface water than in de-ionized water (Fig. 10). This can be attributed to the catalyzing effects of some natural materials present in the natural surface water. The characteristics of natural surface water used in the study are presented in Table 1.

**Table 1:** Characteristics of the natural surface water (Mutale River)

Parameter	Value
pH	6.9
Conductivity (μS/cm)	24
Turbidity (NTU)	14
Sodium (mg/L)	4.66
Calcium (mg/L)	2.15
Potassium (mg/L)	0.35
Magnesium (mg/L)	1.0
SO_4_^2−^ (mg/L)	3.35
Cl^−^ (mg/L)	36.99

### Adsorption isotherm

In this study, the Langmuir isotherm [41] was used to correlate the adsorption equilibrium data obtained. The isotherm is often used to estimate the maximum adsorption capacity corresponding to complete monolayer coverage on the adsorbent surface and is expressed by Eq. (3).

3 $$\frac{1}{{q_{e} }} = \frac{1}{{q_{\text{max} } }} + \left( {\frac{1}{{bq_{\text{max} } }}} \right)\frac{1}{{C_{e} }}$$

where C_e_ is the equilibrium concentration of MB (mg/L), q_e_ is the quantity of MB adsorbed at equilibrium (mg/g), q_max_ is the maximum amount adsorbed (mg/g) and b is the adsorption constant (L/mg). The values of b and q_max_ were obtained from the slope and the intercept of the plots of 1/C_e_ versus 1/q_e_.

The Freundlich isotherm [42] was also used to correlate the adsorption equilibrium data obtained in this work. The linearized form of the Freundlich equation is expressed by Eq. (4).

4 $$\log q_{e} = \log K_{f} + \left( {\frac{1}{n}} \right)\log C_{e}$$

where $q_{e}$ (mg/g) is the adsorption density, $C_{e}$ is the concentration of MB in solution at equilibrium (mg/l) and *K*_*f*_ is the Freundlich constant which relates to the sorption capacity of the adsorbent. Also, the value of $\frac{1}{n}$ indicates the affinity of the adsorbate towards the adsorbent. The experimental data were fitted into Eq. (4) by plotting $logC_{e}$ against $logq_{e} .$ The value of $\frac{1}{n}$ and $logK_{f }$ were determined from the slope and intercept of the plots, respectively.

**Fig. 11:**
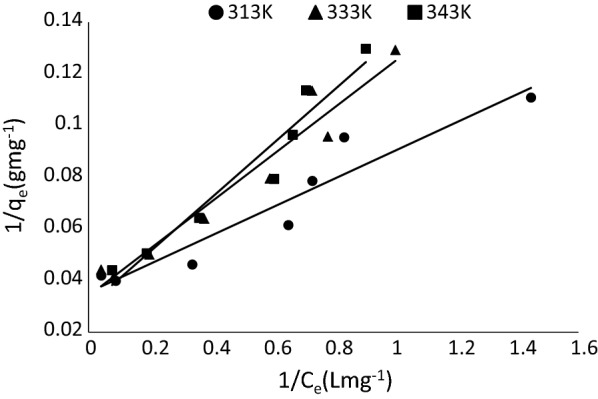
Langmuir plot at different temperatures

From Eq. 3, a plot of 1/Ce versus 1/q_e_ (Fig. 11) gave a straight line with linearized coefficients of 0.91 (313 K), 0.95 (333 K) and 0.94 (343 K), respectively. This implies that the equilibrium data can be described using Langmuir isotherm. The values b and q_max_, are presented in Table 2.

**Table 2:** Langmuir and Freundlich parameters for the adsorption of MB onto Marula seed husk

Temperature	Langmuir isotherm			Freundlich isotherm		
	q_max_ (mg/g)	R_L_ (L/mg)	r^2^	K_f_ (mg/g)	1/n	r^2^
313 K	28.25	0.05	0.92	11.46	0.30	0.80
333 K	29.32	0.08	0.95	9.40	0.34	0.84
343 K	33.00	0.10	0.94	8.64	0.43	0.88

Separation factor (a dimensionless constant) which is an expression of the Langmuir isotherm can also be used to predict if an adsorption system is “favorable” or “unfavorable” by the Langmuir isotherm [43]. This can be evaluated from the relation in Eq. (5);

5 $$R_{L} = \frac{1}{{\left( {1 + bC_{O} } \right)}}$$

where *C*_*o*_ is the initial MB concentration (mg/L) and b the Langmuir constant (L/mg). *R*_*L*_ > 1 indicates an unfavorable monolayer adsorption process, if *R*_*L*_ = 1, the relationship is linear, the process is favorable when 0 < *R*_*L*_ < 1 and if *R*_*L*_ = 0 the process is irreversible. The results obtained from this study has an R_L_ value between zero and one, indicating a favorable adsorption process (Table 2).

**Fig. 12:**
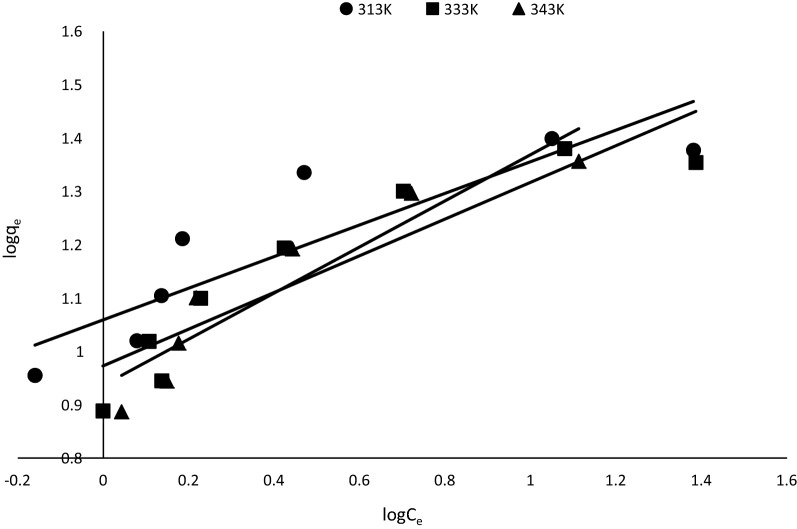
Freundlich plot at different temperatures

A plot of log C_e_ against log q_e_ from Eq. 4 also gave a straight line with linearized coefficients of 0.80 (313 K), 0.84 (333 K) and 0.88 (343 K), respectively (Fig. 12). This also implies that the equilibrium data can also be described by the Freundlich isotherm. However, the Langmuir plot best described the equilibrium data. Tables 2 shows the values of 1/n and K_f_ derived from the slope and intercept of the plot. The values of 1/n were between 0 and 1 indicating that the adsorption of the MB onto the adsorbent used was favorable at the studied conditions [44].

The comparison of the various pulverized adsorbents on MB removal is presented in Table 3. Several factors such as pH, temperature, initial adsorbent dosage, initial MB concentration, nature of the adsorbent and the time of equilibration affect the performance of the adsorbent for the sequestration of MB.

**Table 3:** MB removal capacity of various pulverized seed adsorbent

Adsorbent	q_e_ (mg/g)	pH	Temp (K)	Initial conc (mg/L)	Time (min)	References
Modified Nigella Sativa Seeds	198	11	–	200	30	[45]
Seeds of *Aleurites Moluccana*	42–178	6 and 9	298–328	–	60	[29]
Acid washed Black Cumin seed material	71.94–73.53	7–10	300–318	10–60	30	[40]
Sunflower seed husk (*Helianthus annuus*)	4.76–23.20	4–11	303–353	25–100	–	[46]
Guava seed	0.1	6	298	–	–	[47]
Brazil nut shell	7.81	3–10	303	1100	120	[48]
Mango Seed powder	142.86	8	303	100	–	[49]
Pulverized Marula seed husk	28.25–33.00	5.8	313–343	40	240	This study

### Thermodynamics of the adsorption processes

**Fig. 13:**
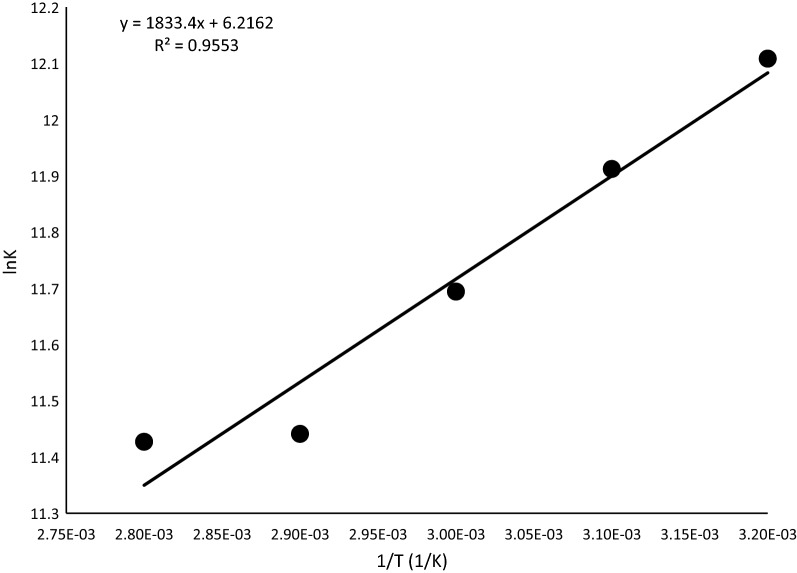
Plot of ln K vs 1/T

The thermodynamic feasibility and the thermal effects of the sorption process were determined by estimating the standard Gibbs free energy change ($\Delta G^{ \circ }$), the standard entropy change $\left( {\Delta S^{ \circ } } \right)$ and the standard enthalpy change ($\Delta H^{ \circ }$). The value of $\Delta G^{ \circ }$ determines if a process occurs spontaneously or not. For a given temperature, a phenomenon is considered to be spontaneous if the $\Delta G^{ \circ }$ has a negative value. Moreover, if $\Delta H^{ \circ }$ is positive, the process is endothermic and if it is negative, the process is exothermic. $\Delta G^{ \circ }$ was determined using the relation in Eq. 6

6 $$\left( {\Delta G^{ \circ } } \right) = - RT\,In\,K_{0}$$

where $K_{0}$ is the equilibrium constant (m^3^ mol^−1^) determined from the Langmuir constant b. $\Delta S^{ \circ }$ and $\Delta H^{0}$ were determined using the Vant Hoff equation (Eq. 7) [50, 51]:

7 $$InK_{0} = \frac{{\left( {\Delta S^{ \circ } } \right) }}{R} - \frac{{\Delta H^{ \circ } }}{RT}$$

where T is the absolute temperature (K) and R is the gas constant, (8.314 J mol^−1^ K^−1^). The plot of $InK_{0}$ as a function of 1/T should give a linear relationship with slope of $\Delta H^{ \circ }$/R and an intercept of $\Delta S^{ \circ }$/R. The values calculated for $\Delta G^{ \circ }$ are presented in Table 4. Figure 13 shows the plot of $InK_{0}$ versus of 1/T; $\Delta S^{ \circ }$ and $\Delta H^{ \circ }$ calculated from the plot are also presented in Table 4.

**Table 4:** Thermodynamic parameters for the adsorption of MB onto *Marula seed husk*

Temperature (K)	$\Delta G^{ \circ }$ (kJ/mol)	$\Delta H^{ \circ }$ (kJ/mol)	$\Delta S^{ \circ }$ (J/mol)
313	− 31.51	15.24	51.68
323	− 31.99		
333	− 32.38		
343	− 32.63		
363	− 34.49

The change in enthalpy $(\Delta H^{ \circ } )$ of the process has a positive value which confirms that the adsorption process is endothermic in nature with the absorption of heat during the sorption process. The negative values of $\Delta G^{ \circ }$ indicate that the process is feasible and spontaneous. The positive values of $\Delta S^{ \circ }$ reflect the affinity of the adsorbents for MB and also suggest an increase in the randomness at the solid/liquid interface during the adsorption of MB onto pulverized Marula seed husk [50, 52]. Similar findings have been reported by Siddiqui et al. [40] and Nayak and Pal [53]. However, a different trend where ΔH and ΔS are negative have also been reported in various studies on the sequestration of MB from aqueous solution [29, 39].

#### Kinetic study of the adsorption processes

The experimental data obtained under the effects of change in time were subjected to three kinetic models (pseudo-first order, pseudo-second order and Elovich kinetic models). Equations 8–10, show the linearized mathematical representation of the models [54, 55], respectively.

8 $$log \left( {q_{e} - q_{t} } \right) = log\,q_{e } - \frac{{k_{1} }}{2.303}t$$

where $q_{e } \left( {mg/l} \right)\;{\text{and}}\;q_{t} \left( {mg/l} \right)$ are the adsorption capacities at equilibrium and at time “t” respectively; $k_{1} \left( {l/min} \right)$ is the pseudo-first-order rate constant. $k_{1} \left( {l/min} \right)$ and $q_{e } \left( {mg/l} \right)$ can then be determined from the slope and the intercept of the plot, respectively.

9 $$\frac{t}{{q_{t} }} = \frac{1}{{k_{2} q_{e}^{2} }} + \frac{t}{{q_{e} }}$$

A plot of $t/q_{t}$ against “t” using Eq. (9) would give a linear relationship from which $q_{e}$ and $k_{2}$ can be determined from the slope and intercept, respectively.

10 $$q_{t} = \frac{1}{\beta } In\left( {\alpha \beta } \right) + \frac{1}{\beta } In\left( t \right)$$

Thus, the plot of $q_{t}$ against $In\left( t \right)$, should give a straight line if adsorption process conforms to Elovich model, where α is the initial adsorption rate (mg/g min); β is the desorption constant (g/mg).

**Fig. 14:**
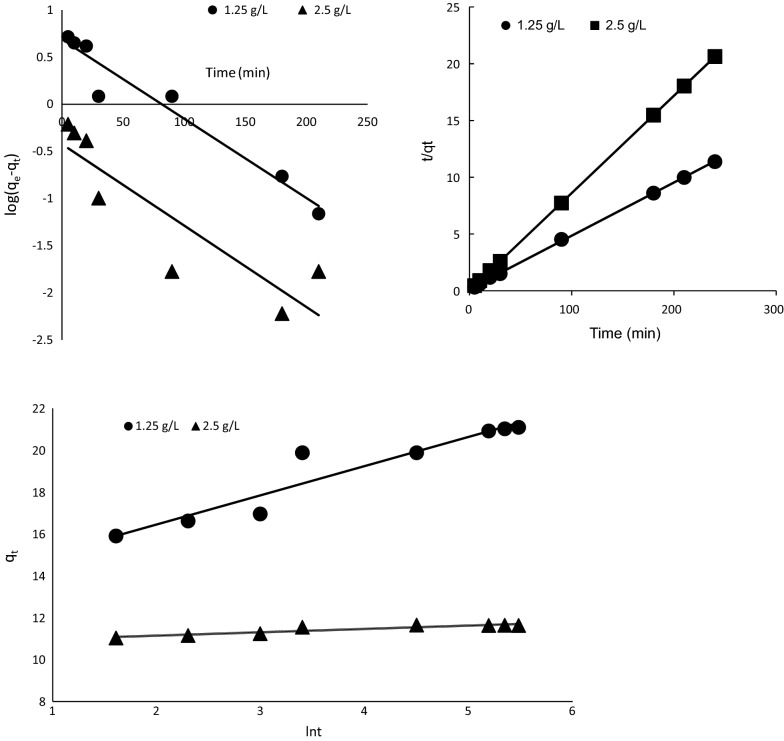
Kinetic plots of the uptake of MB by pulverized Marula seed husk. Top left represents that of the pseudo first order kinetics, top right is for the pseudo second order kinetics while the plot at the bottom represents the Elovich plot

Figure 14 shows the kinetic plots of the three models. The Pseudo second order kinetic best described the kinetics of the adsorption process based on the linearized coefficient. The kinetic constants obtained from the three models are presented in Table 5. Similar results have been reported for MB uptake by various plant materials [52, 56–58].

**Table 5:** Kinetic parameters and constants

Parameter	1.25 g/L	2.5 g/L
Pseudo first order		
R^2^	0.95	0.79
q_e_	4.86	2.65
K_1_	0.0193	0.0298
Pseudo second order		
R^2^	1.00	0.999
q_e_	6.15	30.03
K_2_	0.56	0.01
Elovich		
R^2^	0.91	0.87
β	0.717	6.23
α	10.13	65.57

## Conclusion

The adsorption of MB onto pulverized Marula seed husks was feasible, endothermic and spontaneous from the thermodynamics data evaluated. An optimum pH of 10 was established for the adsorption process. Pseudo second order kinetic model best described the kinetics of the reaction. Although the equilibrium data fitted well into the Langmuir and Freundlich isotherms at various temperatures, the Langmuir isotherm best described the data. Maximum adsorption capacities of 28.25 mg/g (313 K), 29.32 mg/g (333 K) and 33.00 mg/g (343 K) were evaluated.

The adsorption process achieved higher removal efficiency in natural surface water than in de-ionized water. Similarly, base-modified Marula seed husk showed better performance in comparison to the unmodified and acid modified adsorbent. Smaller particle size of the adsorbent favored the process. Marula seed husk can be regarded as a potential candidate for the bioremediation of dyes from aqueous solution and wastewater effluents.

## Abbreviations

α: is the initial adsorption rate (mg/g min)
β: is the desorption constant (g/mg)
b: adsorption energy constant of Langmuir adsorption isotherm (L/mg)
C_e_: equilibrium liquid-phase concentration (mg/L)
C_o_: initial liquid-phase concentration (mg/L)
K_f_: Freundlich isotherm constant related to adsorption capacity [(mg/g) (L/mg) 1/n]
k_1_: rate constant of first-order adsorption (1/min)
k_2_: rate constant of second-order adsorption (g/mg min)
n: Freundlich isotherm constant related to adsorption intensity
Q: the maximum surface coverage (formation of monolayer) of sorbent (mg/g)
q_e_: equilibrium solid-phase adsorbate concentration (mg/g)
qt: amount of adsorption at time t (mg/g)
R^2^: correlation coefficient
R_L_: dimensionless separation factor
V: volume of solution (L)
W: mass of adsorbent (g)
Δ*G*°: standard Gibbs free energy change
Δ*S*°: the standard entropy change
Δ*H*°: standard enthalpy change
MB: methylene blue
t: adsorption time (min)
T: is the absolute temperature (K)
R: is the gas constant
